# Emerging Evidence for the Use of Antidiabetic Drugs, Glucagon-like Peptide 1 Receptor Agonists, for the Treatment of Alzheimer’s Disease

**DOI:** 10.17925/EE.2023.19.1.16

**Published:** 2023-05-23

**Authors:** Ides M Colin, Lidia W Szczepanski, Anne-Catherine Gérard, Jose-Antonio Elosegi

**Affiliations:** 1. Endocrino-Diabetology Research Unit, Department of Internal Medicine, Centre Hospitalier Régional Mons-Hainaut/Groupe Jolimont, Mons Belgium/Groupe Helora, Mons, Belgium; 2. Group of Animal Molecular and Cellular Biology, Louvain Institute of Biomolecular Science and Technology, Université Catholique de Louvain, Louvain-La-Neuve, Belgium; 3. Neurology Unit, Centre Hospitalier Universitaire Ambroise Paré, Mons Belgium/Groupe Helora, Mons, Belgium

**Keywords:** Alzheimer's disease, antidiabetic drugs, glucagon-like peptide 1 receptor agonists, neuronal degeneration, pathophysiological links, type 2 diabetes

## Abstract

From an epidemiological and pathophysiological point of view, Alzheimer’s disease (AD) and type 2 diabetes (T2DM) should be considered 'sister' diseases. T2DM significantly increases the risk of developing AD, and the mechanisms of neuronal degeneration themselves worsen peripheral glucose metabolism in multiple ways. The pathophysiological links between the two diseases, particularly cerebral insulin resistance, which causes neuronal degeneration, are so close that AD is sometimes referred to as 'type 3 diabetes'. Although the latest news on the therapeutic front for AD is encouraging, no treatment has been shown to halt disease progression permanently. At best, the treatments slow down the progression; at worst, they are inactive, or cause worrying side effects, preventing their use on a larger scale. Therefore, it appears logical that optimizing the metabolic milieu through preventive or curative measures can also slow down the cerebral degeneration that characterizes AD. Among the different classes of hypoglycaemic drugs, glucagon-like peptide 1 receptor agonists, which are widely used in the treatment of T2DM, were shown to slow down, or even prevent, neuronal degeneration. Data from animal, preclinical, clinical phase II, cohort and large cardiovascular outcomes studies are encouraging. Of course, randomized clinical phase III studies, which are on-going, will be essential to verify this hypothesis. Thus, for once, there is hope for slowing down the neurodegenerative processes associated with diabetes, and that hope is the focus of this review.

Type 2 diabetes (T2DM) is a disease with a complex aetiopathogenesis that leads to a wide variety of metabolic disorders. This includes, by definition, high plasma glucose levels, but also elevated blood pressure, dyslipidaemia, cardiorenal complications and strokes. All of these disorders, beyond the progressive beta cell failure, derive from insulin resistance (IR).^1^ Among the many T2DM-associated complications, damage to the central and peripheral nervous systems is prevalent in people with T2DM. This can manifest as diabetic peripheral neuropathy (sensory–motor and autonomic) through to cognitive impairment, such as that associated with dementia.^2^ Alzheimer's disease (AD) accounts for 60–80% of dementia cases.^3^ Similarly to other insulin-sensitive organs, including fat, liver and vascular cells, the central nervous system (CNS) is affected by IR, with an increased risk of neuronal degeneration, neuronal death, and structural and functional impairment of the brain.^4^ As a result, cognitive disorders such as those that occur in patients with AD occur more frequently in people with T2DM.^5,6^ Traditional cardiometabolic risk factors, such as a sedentary lifestyle, central obesity, dyslipidaemia, IR, hypertension, diabetes and cardiovascular diseases, are associated with progressive cognitive decline and AD.^5^ According to the Rotterdam Study from 1999, people with T2DM have double the risk of developing AD.^6^

Besides recent interesting discoveries related to the genetic aetiology of AD,^7^ and despite the intensive efforts devoted to AD in the field of pharmacotherapy, no disease-modifying treatments have been demonstrated to adequately stop the progression of AD.^8^ Studies have shown that aducanumab, an amyloid beta (Aβ)-targeting antibody, which was recently approved by the United States Food and Drug Administration (FDA), may have the potential to stop disease progression.^9–11^ However, its ability to modify the outcome of the disease has been disputed.^12^ The decision made by the FDA was based on double-blind, randomized trials that showed a significant dose- and time-dependent reduction in Aβ deposition in participants receiving the active drug.^13^ The treatment was associated with a 22% reduction in cognitive and functional decline. Gantenerumab, another anti-Aβ human immunoglobulin G1 (IgG1) monoclonal antibody, was not as successful. Recently, two clinical trials of gantenerumab showed no significant benefit in patients with early AD (ClinicalTrials. gov identifiers: NCT03444870; NCT03443973).^14–16^ This contrasts with more recent data about lecanemab (Leqembi™; Eisai Co, Ltd., Bunkyo City, Tokyo, Japan), a humanized IgG1 monoclonal antibody, which showed a 27% reduction in cognitive decline compared with placebo, and a decrease in Aβ levels in adults with AD in the phase III Clarity AD trial ( ClinicalTrials. gov identifier: NCT03887455).^17^ Notably, adverse events were more frequently reported in people receiving active treatments, including Aβ-related oedema and effusions, with at least two deaths related to these adverse events.^18^ Consequently, the widespread use of this drug may be questionable. The FDA approved the drug in early 2023.^19^ Approval from the European Medicines Agency followed soon after.^20^

One reason for the poor response of anti-Aβ treatments in AD is the therapeutic target, and especially the accumulation of the missing tau protein, a key protein for neuronal microtubule stabilization and full synaptic activity. Only treatments targeting the double neuropathological process (Aβ deposits and tau accumulation) would be able to limit the progression of the disease. Thus, despite the undeniable efforts and hopes associated with the latest discoveries in the field, the urgency of bringing effective treatments to the market with a real and definitive impact on AD remains paramount. It turns out that certain treatments for T2DM could fill the void by slowing down the neurodegenerative processes associated with diabetes. The aim of this review is to highlight these treatments.

## Alzheimer's disease and type 2 diabetes: a parallel epidemiologic rise

According to the Global Burden of Diseases, Injuries, and Risk Factors Study in 2019, the absolute number of individuals with dementia is estimated to increase from 57.4 million cases worldwide in 2019 to 152.8 million cases in 2050.^21^ This will mainly be due to population growth and population ageing. When age-standardized dementia prevalence is considered, changes in the number of people affected are associated with the increasing prevalence of risk factors, including body mass index (BMI), fasting plasma glucose and smoking.^21^ This is fortunately offset by increases in the average level of education. Therefore, the overall age-standardized prevalence in both sexes will remain fairly stable between 2019 and 2050. There is geographical heterogeneity in the projected increases in AD across countries and regions, with population growth contributing the most to increases in sub-Saharan Africa, and population ageing contributing the most to changes in East Asia.^21^

As emphasized in the 2020 report of the *Lancet* commission, interventions on modifiable risk factors will be a means of coping with the expected increase in the number of individuals with dementia.^22^ Dysregulated glycaemic control is an important risk factor to consider; indeed, evidence suggests that people with T2DM are at a higher risk of developing dementia.^23–26^

According to the latest release from the International Diabetes Federation (IDF), “diabetes is a pandemic of unprecedented magnitude”, with 537 million adults living with diabetes worldwide in 2021, which represents a 16% increase from the previous IDF estimate in 2019.^27^ Even more worrying are the IDF projections of diabetes prevalence, which predict that 783 million adults will be living with diabetes by 2045.^27^ This will represent an increase of 46%, which is more than double the estimated population growth (20%) over the same period.^27^

A growing body of evidence indicates that diabetes increases the risk of dementia by a factor of at least two.^24–26^ A recent study showed that the earlier diabetes develops, the greater the risk of developing dementia.^23^ Cognitive dysfunction can occur in the early stages of diabetes, known as prediabetes.^28^ However, the more the parameters (blood pressure, lipid disorders, waist circumference) of the metabolic syndrome are aggregated, the more the cognitive disorders worsen.^5^ Another study, which used 12-year follow-up data from a populationbased study of Swedish older adults, showed that poorly controlled diabetes (glycated haemoglobin ≥7.5%) is associated with accelerated cognitive deterioration (incident cognitive impairment without dementia and its worsening to dementia).^29^ The association between diabetes and cognitive impairment was stronger when more advanced and severe diabetes was considered (for example, in association with heart disease), suggesting that the worse diabetes is, the greater the risks for advanced brain damage.^29^

In addition to being strongly linked from an epidemiological point of view, these two closely related diseases raise major public health concerns because of the high social and economic burdens they pose on both an individual and a societal level.^24,30^

## Alzheimer's disease and type 2 diabetes: many pathophysiological crosstalks

The pathophysiological mechanisms underlying structural and functional brain damage in individuals with diabetes are multiple and complex.^31^ Strokes, which are part of the many cardiovascular complications associated with diabetes, increase the risk of dementia.^32^ However, they cannot fully explain the association between AD and diabetes, as this cannot be reduced to strokes or hypoglycaemia, and do not seem to provide the most satisfying answer. An alternative explanation could also be that tight glycaemic control with an increased rate of hypoglycaemia leads to brain damage, memory loss and dementia.^33^ However, this explanation is also incomplete.

Rather, accumulated evidence over the last 10–15 years suggests that diabetes-i nduced metabolic disturbances in the brain could alone cause AD.^31,34,35^ AD and T2DM are so close from a pathophysiological point of view that the term 'type 3 diabetes' has even been proposed to describe AD resulting from IR in the brain in order to highlight the strong links between both diseases.^34,36^

### Brain glucose metabolism

**Figure 1:**
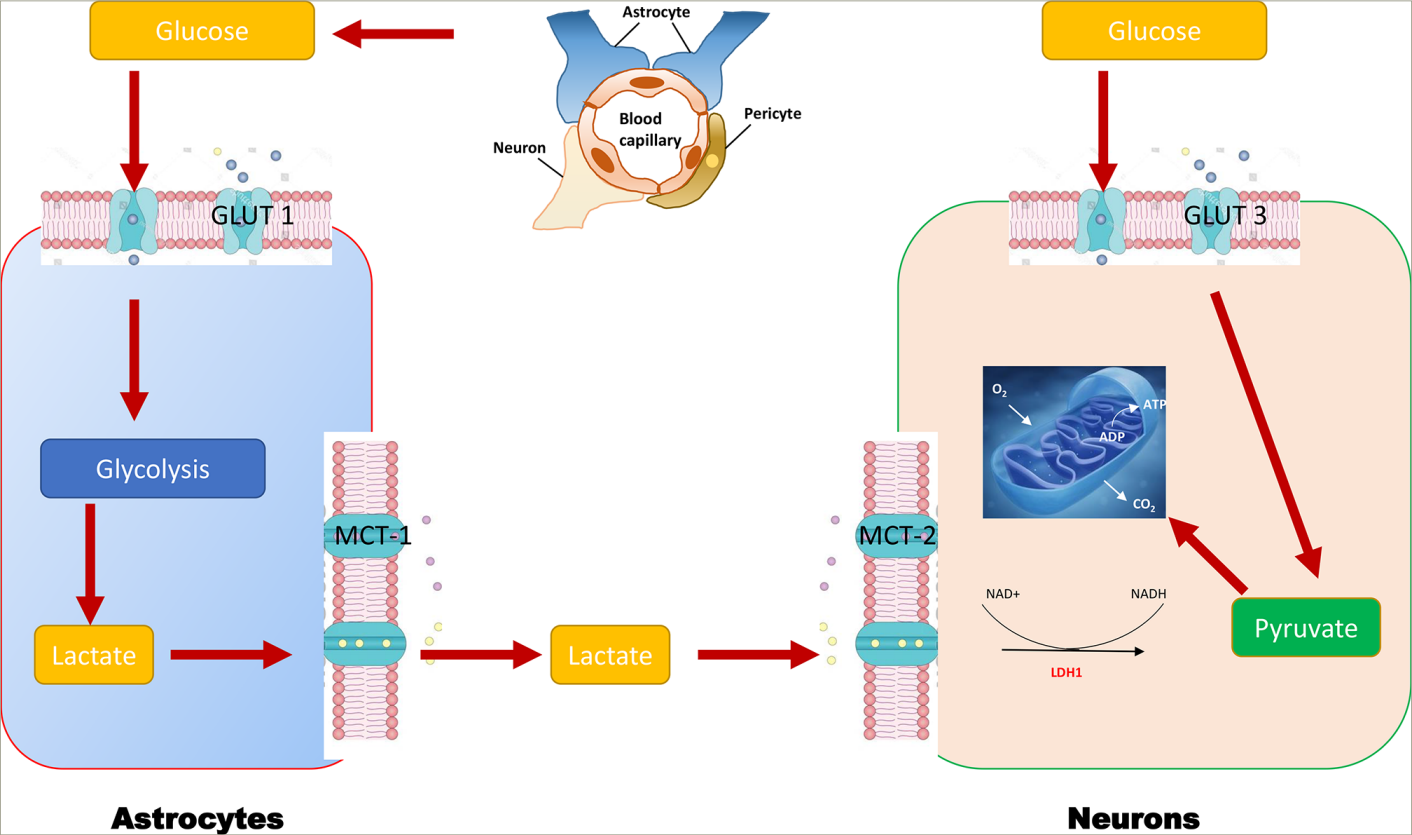
Mechanisms of brain glucose metabolism

Normal glucose metabolism in the brain is essential for preserving neuronal plasticity and neurotrophic and neuroendocrine functions. The brains of people with AD are characterized by decreased cerebral glucose uptake.^37,38^ They produce less energy as provided by glucose and use this energy less efficiently. The body’s main source of energy is glucose; 25% of daily glucose intake is used by the brain, even though this organ represents less than 2% of an individual's total bodyweight.^39^ An uninterrupted supply of glucose is, therefore, essential for normal brain function. Glucose transport across the blood–brain barrier (BBB) is tightly regulated by the glucose transporters (GLUTs) GLUT1 and GLUT3.^39^ In addition, GLUT2 and GLUT4 are present in glial cells, and GLUT4 and GLUT8 are present in neurons.^38^ Along with insulin, the different GLUTs are, therefore, key in the energy environment required for neurons to function normally.^38^ In this context, the role of glial cells is extremely important (*Figure 1*). They sequentially convert glucose into pyruvate during glycolysis and then pyruvate into lactate (the fuel of choice in neurons) through a lactate dehydrogenase-mediated process.^40,41^ Lactate is then taken up into neurons through monocarboxylate transporters, where it is oxidized into pyruvate, which then feeds the tricarboxylic acid cycle to yield adenosine triphosphate (ATP) after oxidative phosphorylation of reduced coenzymes. On the other hand, neurons can capture glucose directly, which feeds the production of ATP through the glycolytic process and the Krebs cycle.^40,41^ To keep normal cognitive functions, the brain’s energetic production must remain optimal and relies on intact insulin signalling in both neurons and glial cells.^42^ Any failure translates inexorably into a loss of cognitive function.

*Glucose is transported from blood vessels to astrocytes through endothelial cells. Astrocytes are specialized in glucose uptake. Glucose transporters (GLUTs) are present in the membrane of glial cells, as well as in neurons. As soon as glucose is transported into astrocytes via GLUT1, it is transformed into lactate through glycolysis. Lactate is then transported outside the astrocyte and taken up by neurons through monocarboxylate transporters. Intracellular lactate is oxidized to pyruvate through lactate dehydrogenase 1 (LDH1) and is metabolized along the oxygen pathway. Glucose may also be transported directly into neurons via GLUT3. Glucose then enters the glycolytic pathway and the tricarboxylic acid pathway for its later oxidation and the production of energy.*

*ADP = denosine diphosphate; ATP = adenosine triphosphate; GLUT = glucose transporter; LDH1 = lactate dehydrogenase 1; MCT = monocarboxylate transporter; NAD+ = oxidized nicotinamide adenine dinucleotide; NADH = reduced nicotinamide adenine dinucleotide.*

### Insulin-induced neuroprotective actions

**Figure 2:**
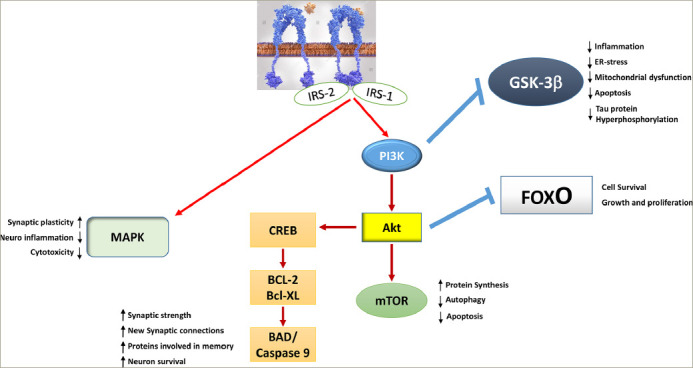
Mechanisms of insulin-i nduced neuroprotective actions

When searching for the common pathophysiological link between AD and T2DM, it is revealed that insulin plays a decisive role. After crossing the BBB via a saturable and thermo-sensitive carrier-mediated active process, insulin binds to its receptor, which triggers an intrinsic tyrosine kinase activity.^43^ The binding of insulin to its receptor induces conformational changes and autophosphorylation of the receptor. This leads to the recruitment and phosphorylation of the insulin receptor substrates IRS-1 and IRS-2 in the neuronal membrane.^44,45^ As a result, the phosphatidylinositol 3-kinase (PI3K)/protein kinase B (Akt) signalling pathway and the extracellular signal-regulated kinase-1/2 mitogen-activated kinase (MAPK) pathway (involved in improved synaptic plasticity and in the regulation of neuroinflammation) are activated. On one hand, Akt inactivates glycogen synthase kinase 3β and forkhead box O; on the other hand, it activates the mammalian target of rapamycin (mTOR), B cell lymphoma 2 (Bcl-2) and B cell lymphoma extra-l arge (Bcl-XL) through cyclic adenosine monophosphate (cAMP) response element-binding protein (*Figure 2*). As a result, insulin exerts beneficial effects in the brain as it positively regulates inflammation-driven processes, endoplasmic reticulum (ER) stress, mitochondrial dysfunction, and apoptosis and promotes mechanisms involved in cell growth and differentiation, and increased synaptic strength, neuronal survival, memory abilities, and learning.^31,34,35,45^ Therefore, when it acts correctly, insulin exerts many benefits in the brain, from long-term neuroprotective and neuromodulatory effects to anorectic activities in the hypothalamus.^46^

### Brain insulin resistance and neuronal degeneration

**Figure 3:**
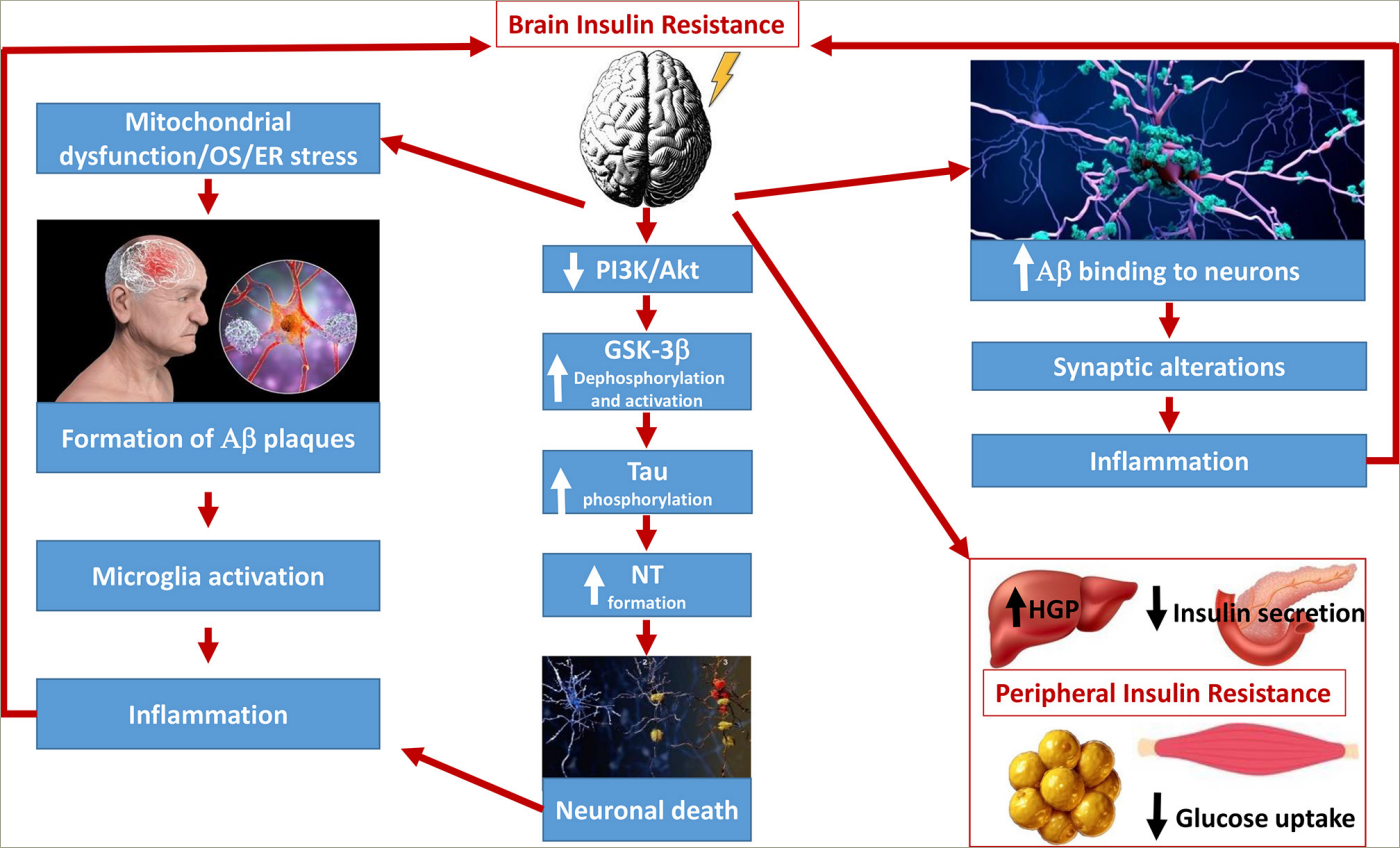
Interplay between brain insulin resistance and neurodegradation

T2DM is characterized by a progressive blunting of insulin sensitivity in the long term that affects the brain, as well as the well-known insulinsensitive organs (e.g. fat cells, hepatocytes, skeletal muscle cells).^47,48^ T2DM increases the risk of developing AD because of brain IR. While IR leads to elevated plasma glucose levels in the periphery, it is responsible for the formation of senile plaques and intracellular neurofibrillary tangles (NTs) in the brain.^49^ Overall, insulin plays an important role in regulating both tau protein, and Aβ deposits in neurons. IR is, for instance, responsible for the hyperphosphorylation of tau, whose abnormal morphological signature is the formation of NTs, which causes axonal transport deficit, neuronal degeneration and neuronal death.^44^ Toxic NTs are known to be associated with decreased expression of GLUT-1 and GLUT-3 in various areas of the brain and mitochondrial dysfunction with increased production of reactive oxygen species.^38,50^ IR also promotes ER stress, which in turn favours tau hyperphosphorylation and IR (in a vicious cycle).^51^ In addition to NTs in neuronal microtubules, senile plaques, which are composed of extracellular Aβ deposits, are another morphologic hallmark of AD.^52^ Insulin regulates the balance between Aβ anabolism and catabolism. The disruption of normal glucose metabolism promotes Aβ aggregation, which, in turn, worsens the neuronal IR in a vicious cycle.^48^ The number of dysfunctional neurons slowly increases, both due to intracellular protein aggregates and the accumulation of senile plaques with undigested Aβ peptides. In a vicious cycle, these anomalies could also be upstream mechanisms of mitochondrial dysfunction with increased reactive oxygen species production, as well as dysfunction of mitophagy and autophagy. These processes are determinant as they contribute to further neuronal degradation and irreversible progression of AD in T2DM.^31,34,35,47–49^ In addition, the synaptic transmissions are impaired due to the decrease in production of acetylcholine, the main neurotransmitter in memory processes (cognition), due to the lack of metabolites produced in the glycolytic pathway (coenzyme A and succinyl coenzyme A) (*Figure 3*).^46,53,54^ Diabetes-i nduced micro-and macrovascular changes, disruption of the BBB, and increased local inflammation should also be considered in this complex pathophysiological interplay.^34,35,55^ Thus, together with Aβ deposition, the local microinflammation derived from microglia activation is responsible for defective neurogenesis and reduced neuronal survival, for instance, in defective hippocampal-dependent memory.^56,57^

*Insulin-induced phosphorylation of insulin receptor substrate activates phosphoinositide-3 kinase (PI3K)/protein kinase B (Akt) and mitogen-activated kinase (MAPK) pathways. PI3K/ Akt pathway then inactivates glycogen synthase kinase 3β and forkhead box O and activates mammalian target of rapamycin. Altogether, insulin sensitive-dependent intracellular pathways regulate many mechanisms involved in cell survival, proliferation, apoptosis, protein synthesis, inflammation, endoplasmic reticulum stress, autophagy and mitochondrial function. Through cyclic adenosine monophosphate response element-binding protein, Akt also stimulates B cell lymphoma 2, B cell lymphoma extra-large and B cell lymphoma 2 antagonist of death, which regulates learning, memory and neuron survival. MAPK influences mechanisms involved in cell proliferation, differentiation, apoptosis or survival, and neuroinflammation.*

*Akt = protein kinase B; BAD = Bcl-2 antagonist of death; Bcl-2 = B cell lymphoma 2; Bcl-XL = B cell lymphoma extra-large; CREB = cyclic adenosine monophosphate response element-binding protein; ER = endoplasmic reticulum; FoxO = forkhead box O; GSK-3β = glycogen synthase kinase 3β; IRS = insulin receptor substrate; MAPK = mitogen-activated kinase; mTOR = mammalian target of rapamycin; PI3K = phosphoinositide-3 kinase.*

Although dominant for over three decades, the hypothesis that Aβ cascade is directly responsible for AD is being challenged strongly.^58^ Aside from fraudulent research, as recently revealed in *Science*,^59^ it is also known that many people with no clinical manifestations of dementia actually have neuropathological hallmarks of AD.^58^ Moreover, from the analysis of 14 anti-Aβ drug clinical trials, targeting Aβ and tau does not appear to improve cognitive functions in people with AD.^60^

Given the intimate pathophysiological links between AD and T2DM, there is growing interest in using drugs that have a positive impact on cardiorenal complications for more neurological purposes.^61^

## Antidiabetic drugs in Alzheimer's disease: two diseases but one therapeutic target

### Lifestyle intervention

Upstream of drug intervention, non-pharmacological and lifestyle interventions for individuals with AD include the following:

- education
- social engagement
- cognitive stimulation
- smoking cessation
- exercise
- management of depression and psychological stress
- management of cerebrovascular disease
- management of hypertension
- management of dyslipidaemia
- management of T2DM
- management of obesity
- a healthy diet.

Beyond improving metabolic control, these interventions can have positive effects in terms of quality of life, cognitive decline and incidence of AD, as has recently been reported.^62–64^ Lifestyle interventions should, therefore, always be recommended for all patients with T2DM and/or AD, as they produce beneficial effects on IR and have positive impacts on various biological markers of AD.^65^

Among the various therapeutic tools available for the treatment of diabetes, insulin itself might appear, at first glance, to be a valid option. It is true that the systemic administration of insulin sometimes improves cognitive functions.^66^ However, given the narrow therapeutic threshold of the treatment, the inherent hypoglycaemic risk and the consequences in terms of the resulting cognitive dysfunction, insulin should always be used cautiously in this indication.^67,68^ There have also been clinical trials with intranasal insulin that have shown inconsistent results in improving cognitive functions.^69–71^

*In the case of insulin resistance, glycogen synthase kinase 3β is permanently dephosphorylated and therefore activated, thereby hyperphosphorylating tau, which contributes to the formation of neurofibrillary tangles and neuronal death. Mechanisms linked to beta-amyloid deposits are also upregulated, leading to synaptic alterations. The resulting neuroinflammation, in turn, aggravates insulin resistance in a vicious circle. In addition, the neuronal degeneration that affects the hypothalamus fuels impaired regulation of peripheral metabolism, with insulin resistance affecting insulin-sensitive organs such as adipocytes and skeletal muscle cells while impairing insulin secretion by pancreatic beta cells and the distribution of the peripheral fat.* ^53^

*Aβ = beta-amyloid; Akt = protein kinase B; ER = endoplasmic reticulum; GSK-3β = glycogen synthase kinase 3β; HGP = hepatic glucose production; NT = neurofibrillary tangle; OS = oxidative stress; PI3K = phosphoinositide-3 kinase.*

### Pharmacological intervention

Evidence regarding the positive effects of metformin on cognitive impairment is lacking. Although studies in rodents suggest that metformin may improve cognitive functions, a study on the risk of AD in humans showed that metformin treatment is instead associated with poorer cognitive performances.^72,73^ In contrast, a meta-analysis by Campbell et al. showed that metformin users have a lower risk of AD,^74^ while another by Tabatabaei Malazy et al. showed that metformin does not significantly improve cognitive functions.^75^ Regarding AD, the place of metformin, therefore, remains controversial.

The evidence regarding sulphonylureas is clearer: they are associated with worsening cognitive deficits. Recent data by Tang et al. from nationwide electronic medical records from the US Veterans Affairs Healthcare System showed a 12% increased risk of all-cause dementia with sulphonylurea monotherapy compared with metformin monotherapy.^76^ The hazard ratio (HR) for all-cause dementia for sulphonylurea monotherapy was even higher in people with a high BMI (HR 1.15, 95% confidence interval [CI] 1.10–1.21 for individuals with BMI >25 kg/m^2^ versus HR 1.02, 95% CI 0.93–1.12 for individuals with BMI <25 kg/m^2^ [2-year exposure]; p<0.01).^76^

In contrast, several studies have reported improved cognitive function in people treated with thiazolidinediones (TZDs). The large real-world study by Tang et al. further reinforced the idea that TZDs protect against dementia.^76^ TZDs were associated with a lower risk of AD (HR 0.89; 95% CI 0.86–0.93) compared with metformin monotherapy.^76^ In combination with metformin, TZDs further reduced the risk of allcause dementia, particularly in younger people (below 75 years) and in those with a BMI greater than 25 kg/m^2^.^76^ Positive effects are likely restricted to pioglitazone, which has good BBB penetration, in contrast with rosiglitazone, which has poor BBB penetration.^77^ A recent phase III randomized controlled trial (the TOMMORROW trial; ClinicalTrials. gov identifier: NCT01931566), however, failed to show the efficacy of pioglitazone (versus placebo) in delaying the onset of mild cognitive impairment in a population of at-risk participants without diabetes over a period of 5 years.^78^ Ten years earlier, a randomized pilot clinical study also failed to show positive improvement in cognitive function in people with AD who did not have diabetes, who were treated with pioglitazone for 18 months.^79^

Sodium-glucose co-transporter 2 (SGLT2) inhibitors are now widely used for treating T2DM, especially in people with cardiorenal complications.^80^ Their use is associated with reduced risk of dementia in people with T2DM; data from the recent nested case-control study by Wium-Andersen et al. indicate that this class of drug actually has neuroprotective effects.^81^ The study enrolled 176,250 people with T2DM registered in the Danish National Diabetes Register. Using risk-set sampling, each dementia case (n=11,619) was matched on follow-up time and calendar year of dementia, with four controls randomly selected among cohort members without dementia (n=46,476). Antidiabetic medications were categorized into the following types: insulin, metformin, sulphonylureas and glinides combined, TZDs, dipeptidyl peptidase-4 (DPP-4) inhibitors, glucagon-like peptide 1 (GLP-1) receptor agonists (RAs), SGLT2 inhibitors, and acarbose.

Use of metformin, DPP-4 inhibitors, GLP-1 RAs and SGLT2 inhibitors were associated with lower risk of dementia, with a gradual decrease in risk of dementia for each increase in daily defined dose. There were no synergistic effects of combined treatments.^81^ This is in accordance with a more recent study, which reported that SGLT2 users have a lower incidence of dementia compared with those on DPP-4 inhibitors.^82^ It is, therefore, likely that SGLT2 inhibitors may find some room in maintaining cognitive functions in people with T2DM.^61^ A double-blind, randomized placebo-controlled, parallel-group, 12-week study of the effects of dapagliflozin in people with AD is underway ( ClinicalTrials. gov identifier: NCT03801642).^83^

A recent study by Akimoto et al. used a using a model of logistic regression to show that rosiglitazone, exenatide, liraglutide, dulaglutide and sitagliptin have a significantly lower associated risk of AD than metformin.^84^ Among these different drugs, GLP-1 RAs appear to have the largest potential to improve cognitive performance.

## Glucagon-like peptide 1 receptor agonists: a place in the treatment of Alzheimer's disease?

### Glucagon-like peptide 1 receptor agonists: what are they?

GLP-1 (7-36) amide is an incretin hormone (a 30-amino acid peptide hormone) that is continuously released by enteroendocrine L cells in the small intestine but is released in greater amounts in response to food intake.^85^ Its main physiological actions include the ability to enhance glucose-dependent insulin secretion (the incretin effect), suppress postprandial glucagon secretion, slow gastric emptying and induce satiety through hypothalamic stimulation. Native human GLP-1 exhibits a short half-l ife of less than 2 minutes due to the rapid cleavage by an ubiquitous aminopeptidase, DPP-4, and renal clearance. Long-acting proteolysis-resistant GLP-1 RAs have, therefore, been engineered for treating T2DM and obesity.^86^ The first GLP-1 RA was approved in 2005 for the treatment of T2DM.^87^ Since then, different GLP-1 RAs, classified on their molecular backbone derived either from native human GLP-1 or from exendin-4 (a salivary gland hormone from the Gila monster lizard, *Heloderma suspectum*) have been developed. The human GLP-1 analogues include the following:

- liraglutide, which has 97% sequence homology to human GLP-1, with two amino substitutions and a fatty-acid side chain that enables albumin binding (once daily, with a half-l ife of 13 hours);
- semaglutide, which has 94% sequence homology to human GLP-1, with the addition of a fatty diacid chain and a spacer and two amino acid substitutions (once weekly, with a half-l ife of 165–185 hours); and
- dulaglutide, which contains two chains of human GLP-1 covalently linked to Fc fragment of human IgG4, bound via a small peptide linker (once weekly, with a half-l ife of 90 hours).

The exendin-4 analogues include the following:

- exenatide, which has 53% sequence homology to human GLP-1 (twice daily, half-l ife of 2.4 hours); and
- lixisenatide, which has 50% sequence homology to human GLP-1 (once daily, with a half-l ife of 3 hours).

In addition, a novel GLP-1/glucose-dependent insulinotropic polypeptide co-agonist has been developed for the treatment of T2DM and obesity.^86^ Beyond providing effective glycaemic control, along with weight reduction and lower risk of hypoglycaemia, GLP-1 RAs have been shown to have safety and tolerability profiles that confer long-term beneficial effects on cardiovascular outcomes in people with T2DM.^85,88^ GLP-1 RAs occupy a prominent place in the 2023 therapeutic recommendations by the American Diabetes Association and the European Association for the Study of Diabetes.^85,88^

The latest generation of GLP-1 RAs, such as semaglutide, reduce energy intake by decreasing appetite and increasing satiety through the direct activation of the hypothalamus and hindbrain and the indirect activation via the vagus nerve.^89^ However, beyond these now well-accepted positive metabolic effects, GLP-1 RAs are also known to improve cognition.^90–92^ The role played by GLP-1 as a neurotransmitter was already reported in 1996.^93^ This class of drugs has shown neuroprotective effects in preclinical studies as they improve memory and learning and prevent Aβ depositions and the formation of NTs.^90–92,94^

### Glucagon-like peptide 1 receptor agonists: how can they be neuroprotective?

The mechanism by which GLP-1 RAs exert neuroprotective effects is complex. Once cleaved from its (pre)proglucagon, GLP-1 acts on its receptors (GLP-1 receptor, a 7-transmembrane class B1 G-coupled receptor family). Besides peripheral tissues, including the gut, stomach, pancreas, kidneys, heart, adipose cells, bones and blood vessels, GLP-1 receptors are also expressed in the CNS, along with the GLP-1 ligand.^86^ Thus, preproglucagon-expressing neurons are found in the nucleus tractus solitarii of the brainstem and send projections to the arcuate and paraventricular nuclei of the hypothalamus.^91^ These neurons in the nucleus tractus solitarii are directly activated by afferent vagal inputs to relay satiety signals from the periphery to the brain. However, there are many other cerebral regions (cortex, hippocampus, striatum, substantia nigra) where preproglucagon-producing cells are expressed.^86,91^ GLP-1 RAs have a widespread distribution in the CNS, from the occipital and frontal lobes to the hypothalamus and thalamus, the caudate putamen, globus pallidus and hippocampus.^86,91,95^ In the case of T2DM and obesity-related IR, impaired GLP-1 secretion contributes to neuronal degeneration and cognitive decline, whereas the administration of exogenous GLP-1 RAs reverses these pathogenic changes.^90^ To act in the brain, GLP-1 and GLP-1 RAs cross the BBB by simple diffusion.^91,96^ This process has been shown, at varying rates, with exendin-4 (the bioactive peptide derived from *H. suspectum* venom) and exenatide (the pharmacologic mimetic), which exhibit a good penetration rate, followed by lixisenatide and finally, the lipidated peptides, liraglutide and semaglutide.^90,91^ They reach various areas in the CNS where they exert anorectic effects, in addition to acting as anti-i nflammatory and neuroprotective agents.

**Figure 4:**
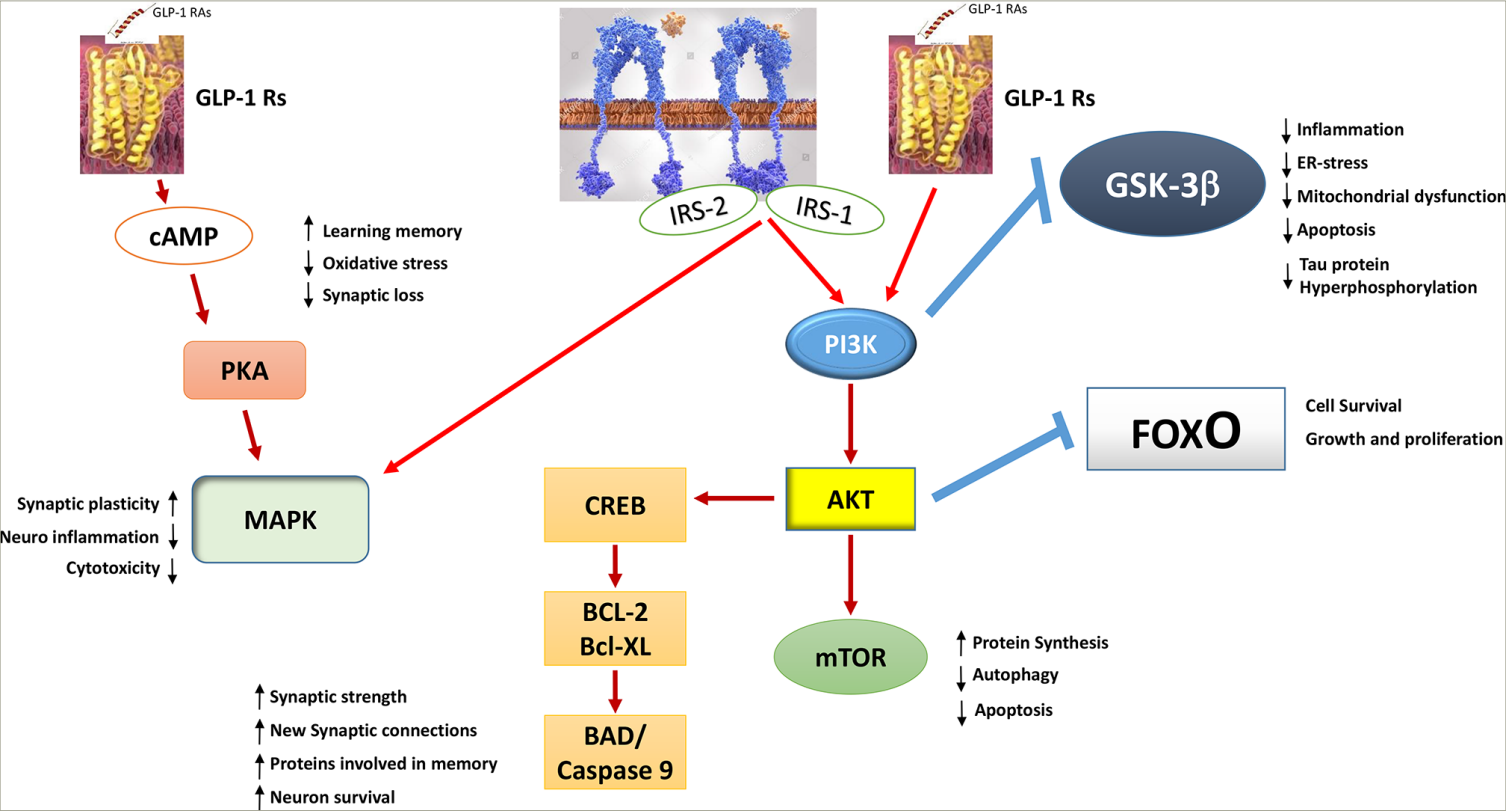
The overlapping glucagon-like peptide 1 signalling and insulin signalling pathways in neurons

As soon as GLP-1 binds to its receptor, intracellular adenylyl cyclase is activated; this, in turn, increases cAMP levels. Protein kinase A/ PI3K pathways are then activated. As downstream intracellular pathways common to insulin, including MAPK, are activated by GLP-1, it is now thought that GLP-1 RAs actually restore insulin-dependent intracellular altered pathways through activated overlapping pathways (*Figure 4*).^86,90–92,94^ As GLP-1 also prevents the loss of insulin brain receptors, which is a common feature of AD, it definitively acts as a neuroprotective agent.^97^ This neuroprotective effect was reported for the first time more than 20 years ago, and the physiological role of GLP-1 in cognition was reported in the following years.^98,99^ For instance, GLP-1 receptor-deficient mice that have a phenotype characterized by a learning deficit had this deficit restored after hippocampal *GLP1R* gene transfer.^99^

*The activation of glucagon-like peptide 1 (GLP-1) receptors activates protein kinase A (PKA) through adenosine cyclase and increased intracellular cyclic adenosine monophosphate levels. The downstream common pathways with insulin are phosphoinositide-3 kinase (PI3K) and mitogen-activated kinase pathways. The activation of GLP-1 receptors, therefore, increases insulin sensitivity and compensates for impaired insulin signalling. Tau hyperphosphorylation is reduced along with neurofibrillary tangle and beta-amyloid deposits.*

*AKT = protein kinase B; BAD = BCL-2 antagonist of death; BCL-2 = B cell lymphoma 2; Bcl-XL = B cell lymphoma extra-large; cAMP = cyclic adenosine monophosphate; CREB = cyclic adenosine monophosphate response element-binding protein; ER = endoplasmic reticulum; FoxO = forkhead box O; GLP-1 RAs = glucagon-like peptide 1 receptor agonists; GSK-3β = glycogen synthase kinase 3β; IRS = insulin receptor substrate; MAPK = mitogen-activated kinase; mTOR = mammalian target of rapamycin; PI3K = phosphoinositide-3 kinase; PKA = protein kinase A.*

GLP-1 RAs act on many other intracellular pathways involved in neuroinflammation and neuronal degeneration. They restore altered mitochondrial dysfunction, such as in the hippocampus. They also contribute to the decrease of Aβ deposits and tau hyperphosphorylation, and they restore synaptic losses.^86,90–92,94^ The method of action of GLP-1 RAs is, therefore, complex and involves direct mechanisms, including improved mitochondrial function, reduced ER stress and neuroinflammation, and indirect mechanisms including reduced blood glucose levels and, more importantly, improved IR.

### Evidence from animal experiments

Several animal studies has shown that liraglutide decreases Aβ and NT depositions, and has a positive influence on cognitive function. In a transgenic mutant tau (hTauP301L) mouse tauopathy model, liraglutide was shown to reduce neuronal phospho-tau load significantly compared with the vehicle-dosed controls.^100^ In this model, liraglutide treatment significantly reduced the clasping rate (a measure of hind limb motor impairment) and fully prevented clasping-associated lethality.^100^ In addition, liraglutide improved memory function in a model of senescence-accelerated mouse-prone 8 (SAMP8) mice, which naturally occurrs in mice that displays a phenotype of accelerated ageing.^101^ In this model, liraglutide delayed the age-associated progressive decline in spatial memory function associated with hippocampal neuronal loss. Compared with age-matched vehicle-dosed SAMP8 mice, liraglutide also significantly increased the number and the density of *cornu ammonis* 1 (CA1; Latin for 'horn shaped)' pyramidal neurons, which appear to be critical for object differentiation in long-term memory. Hippocampal neurons are, therefore, preserved in these mice with AD treated with liraglutide.^101^ Other preclinical studies showed that liraglutide prevents key neurodegenerative developments found in AD.^90,92^

### Evidence from clinical trials

In a pilot study where liraglutide was administered in people with longstanding AD (n=18), no difference in terms of cognition or Aβ deposition was found compared with those on placebo (n=20) after 26 weeks.^102^ The individuals in this clinical assay were not diabetic. Notably, 26 weeks of liraglutide treatment prevented the expected decline in the cerebral metabolic rate of glucose (measured by [^18^F]fluorodeoxyglucose-positron emission tomography, a tracer of brain metabolic changes), which reflects disease progression. This finding may be associated with improved BBB glucose transport in people treated with liraglutide.^102^ More recently, a randomized controlled clinical study carried out in a group of people with obesity, prediabetes or early T2DM (n=20 in each arm) showed that, after comparable weight loss and superimposable glycaemic control and insulin sensitivity, liraglutide (1.8 mg daily administered subcutaneously) significantly improved short-term memory (mean Digit Span z-score: from -0.06 to 0.80; p=0.024) and memory composite z-score (mean memory z-score: 0.67 to 0.032; p=0.0065).^103^ Data from pooled doubleblind, randomized controlled trials (15,820 patients) indicated that people treated with a GLP-1 RA had a lower rate of dementia than people on placebo (HR 0.47, 95% CI 0.25–0.86).^104^ These data are in accordance with a *post hoc* analysis of the cardiovascular outcome REWIND trial ( ClinicalTrials. gov identifier: NCT01394952), which showed that dulaglutide reduces cognitive impairment by 14% in people with T2DM aged 50 years or above who had additional cardiovascular risk factors; these results were seen during a median follow-up of 5.4 years.^105^ Data from a nationwide Danish registry-based cohort (120,054 patients) also showed that dementia rate was lower in those treated with GLP-1 RAs compared with placebo (HR 0.89, 95% CI 0.86–0.93), with yearly increased exposure to GLP-1 RAs.^104^ A placebo-controlled, double-blind, phase II clinical trial (the ELAD trial; ClinicalTrials. gov identifier: NCT01843075) testing liraglutide in over 200 patients with mild cognitive impairments/AD for 1 year showed that neuronal loss was reduced by the drug.^106,107^ Two phase III clinical trials testing semaglutide in patients with AD are currently on-going: the EVOKE trial ( ClinicalTrials. gov identifier: NCT04777396)^108^ and the EVOKE Plus trial (ClinicalTrials. gov identifier: NCT04777409).^109^ These two trials will study the efficacy of oral semaglutide (14 mg) in patients with early AD. Change in clinical dementia rating, time taken to reach dementia and change in the AD Composite Score are some of the important outcomes that will be assessed from these trials.^108–110^

## Conclusions

Currently, GLP-1 RAs belong to a class of drugs that are used extensively for treating T2DM. Their place in the treatment of T2DM has been further reinforced in the latest version of the treatment guidelines published jointly by the American Diabetes Association and the European Association for the Study of Diabetes.^85,88^ In addition to T2DM, GLP-1 RAs have been emerging as a potential treatment for AD for well over a decade.^35^ Both diseases, whose worldwide prevalence continues to rise, are now recognized as having particularly serious consequences in socioeconomic terms and severely impact both the quality of life and morbi-mortality of people who are affected.^21,27^ Beyond the usual micro-and macrovascular complications associated with T2DM, one must admit that the strong relationship between diabetes and neuronal degeneration remains somewhat evanescent among diabetologists. According to accumulated evidence over the last 10 years, this relationship is, in fact, relevant and logical. AD and T2DM share common pathophysiological mechanisms, namely IR, which led some to claim that AD is a metabolic disease caused by IR in the brain, thereby supporting the hypothesis that AD should actually be regarded as type 3 diabetes.^34,36,111^

Early 2023 was marked by the announcement made by the FDA to recognize lecanemab as an effective treatment for AD.^19^ In addition, the European Medicines Agency has accepted a marketing authorization application for lecanemab.^20^ There are undeniably great expectations from the medical community and health authorities with regard to the marketing of innovative molecules. The future in the field also lies in the refinement of screening methods. Early diagnosis of AD using the biomarker called 'brain-derived tau' (BD-tau) will likely bring additional value to this topic.^112^ The test is presented to be superior to current blood diagnostic tests that are usually used to detect AD. It is specific to the disease and correlates well with AD neuronal degeneration biomarkers in the cerebrospinal fluid. The technique was developed to selectively detect BD-tau while avoiding misleading contaminants produced outside the brain. BD-tau plasma levels correlate with those in the cerebrospinal fluid, as well as with the severity of Aβ plaques and NTs in the brain tissue. Early detection of the disease is particularly sought since it would trigger the adoption of preventive methods, in particular those associated with improved insulin sensitivity at the cerebral level. It is important to keep in mind that, although attractive, the hypothesis that GLP-1 RAs could play a role in the treatment of AD remains to be demonstrated and clinical trials are still on-going. If these trials could demonstrate the efficacy of GLP-1 RAs (as preclinical and phase II studies seem to suggest) as a relevant treatment of AD, this would undoubtedly represent a breakthrough and an enormous hope, since their use is widespread and well known, in particular to diabetologists.
